# Urban-rural disparity in sociodemographic characteristics and sexual behaviors of HIV-positive adolescent girls and young women and their perspectives on their male sexual partners: A cross-sectional study in Zimbabwe

**DOI:** 10.1371/journal.pone.0230823

**Published:** 2020-04-23

**Authors:** Ibou Thior, Elizabeth Rowley, Webster Mavhu, Natalie Kruse-Levy, Lyn Messner, Zachariah J. Falconer-Stout, Owen Mugurungi, Getrude Ncube, Suzanne Leclerc-Madlala

**Affiliations:** 1 PATH, Washington, D.C, United States of America; 2 Centre for Sexual Health & HIV/AIDS Research, Harare, Zimbabwe; 3 U.S. Agency for International Development (USAID), Harare, Zimbabwe; 4 EnCompass LLC, Rockville, Maryland, United States of America; 5 AIDS and TB, Ministry of Health and Child Care, Harare, Zimbabwe; 6 USAID, Arlington, Virginia, United States of America; Kenya Medical Research Institute, UNITED STATES

## Abstract

We conducted a cross sectional survey in Zimbabwe to describe urban-rural disparity in socio-demographic characteristics and sexual behaviors of HIV-positive adolescent girls and young women (AGYW) and their male sexual partners. Between September and November 2016, we interviewed 360 sexually active HIV positive AGYW, aged 15––24 years attending ART and PMTCT clinics in urban and rural health facilities in Harare and Mazowe district respectively. HIV positive AGYW in rural areas as compared to those in urban areas were older, less educated, more frequently married or cohabiting, had lower number of male sexual partners in their lifetime and in the last 12 months preceding the survey. They were mostly heterosexually infected, more likely to disclose their status to a family member and to be more adherent to ART (OR = 2.5–95% CI = 1.1–5.5). Most recent male sexual partners of HIV positive AGYW in urban areas as compared to those from rural areas were mainly current or former boyfriends, single, more educated, less likely to have a child with them and to engage in couple voluntary counseling and testing (CVCT). They were more likely to patronize dancing and drinking venues and involved in transactional sex (OR = 2.2–95% CI: 1.2–4). They were also more likely to be circumcised (OR = 2.3–95% CI: 1.3–4.1) and to use condom more consistently in the last 12 months preceding the survey. Our study findings called for the strengthening of HIV prevention interventions in urban areas among HIV positive AGYW who had more than one partner in their lifetime or are patronizing dancing and drinking venues. In Zimbabwe, promotion of CVCT, index testing, male circumcision and condom use should be sustained to engage male sexual partners of both urban and rural HIV positive AGYW in HIV prevention.

## Introduction

According to a report by the Joint United Nations Programme on HIV/AIDS (UNAIDS), every week 6,200 young women aged 15–25 years become infected with HIV. The same report showed that more than half of all new infections in 2018 occurred in sub-Saharan Africa [1].

In Zimbabwe, females of this age range have a disproportionately higher HIV prevalence compared to their male counterparts. The most recent Zimbabwe Demographic and Health Survey (ZDHS) findings showed that among females, HIV prevalence increases steadily with age, from 2.7% of women aged 15–17 to 13.9% of those aged 23–24 [2]. Among young men, HIV prevalence holds steady at around 2.5% until the age of 23–24, when it increases to 6.0% [2]. Age-disparate sex, multiple partnership, transactional sex, early sexual debut, social and gender norms, and gaps in knowledge have been identified as factors that contribute to HIV infection among adolescent girls and young women (AGYW) in sub-Saharan Africa (SSA) and Zimbabwe in particular [3–9]. The identification of these important determinants of HIV transmission led to several studies aimed at describing the socio-demographic characteristics and sexual behaviors of male partners of AGYW and implementing more targeted interventions [10, 11].

A study describing the male sexual partners of AGYW in Kenya showed several disparities between those from rural and urban settings [10]. Rural AGYW achieved lower levels of education and were less likely to be currently in school. They were more likely to be married or living as married as compared to their urban peers. Among AGYW who reported having had sex in the past 12 months, those from urban areas (16%) reported more multiple partners than their rural peers (0.7%). Male partners of rural AGYW in the past 12 months preceding the survey were more likely to be older (76%) than those of urban AGYW (8%). There were more medically circumcised male partners in urban areas (92%) than in rural areas (67%).

Most HIV-positive AGYW in Zimbabwe are infected sexually and continue to engage in sexual relationships after infection [12–15]. In addition, HIV prevalence among adolescents in Zimbabwe also has been partially attributed to long-term survivors of mother-to-child transmission [16, 17]; and these perinatally infected adolescents may engage in risky sexual behaviors [18, 19]. Positive changes in sexual behavior before and after seroconversion in southern African women, including Zimbabwean women, have been reported; however, these changes have been shown to be modest (less than 10% change in the frequency of sexual risk behavior) and warrant additional interventions [14, 20, 21]. Lack of disclosure of HIV status to sexual partners, inconsistent condom use, multiple concurrent sexual partnerships, and suboptimal adherence to antiretroviral therapy (ART) have been described as risky behaviors among HIV positive AGYW and their sexual partners [22–31]. Adherence to ART is one of the greatest challenges for improving health outcomes among adolescents living with HIV. ART has immediate health and HIV prevention benefits. ART adherence has been challenging because of multiple factors including emotional and lifetime characteristics, cultural and social factors and health services related issues [32–37]

There are limited comparative analyses of male sexual partners and sexual behaviors of HIV-positive AGYW from urban and rural areas of Zimbabwe. This study sought to describe the urban-rural disparity in socio-demographic characteristics, male sexual partnership and risk behaviors among HIV-positive AGYW aged 15–24 in Zimbabwe. Understanding those disparities could better inform intervention and prevention activities for AGYW living with HIV and their male sexual partners.

## Methods

### Study design and setting

A quantitative cross-sectional survey from a mixed-methods approach was conducted to describe the sociodemographic characteristics of HIV-positive AGYW and key aspects of their sexual behaviors, with an emphasis on the background and relationship characteristics of their most recent male sexual partners in the 12 months preceding the survey. The results of the qualitative survey have been described elsewhere [30]. Participants were recruited from four urban health facilities offering ART and prevention of mother-to-child transmission (PMTCT) services to HIV-positive AGYW in Harare, Zimbabwe's capital city, and in two rural health facilities in Mazowe district, Mashonaland Central Province. In Harare, participants were recruited from Parirenyatwa hospital (from an outpatient clinic with a cohort of HIV positive AGYW) and three health centers located in downtown and outskirts of Harare, respectively. Mazowe study site (Concession hospital and one additional clinic) was selected among rural areas because of its proximity to Harare and it is one of the DREAMS (Determined, Resilient, Empowered, AIDS-free, Mentored and Safe) focus sites in Zimbabwe. DREAMS is a public-private partnership that aims to reduce HIV infections among AGYW in 10 sub Saharan Africa countries through a multipronged approach that includes mobilizing communities, access to combination prevention, the engagement of men, integration of HIV services into sexual and reproductive services, and efforts to keep girls in school. AGYW were recruited into the study if they were aged 15–24 years, aware of their HIV-positive status (status confirmed by clinic record and/or enrollment in ART or PMTCT program), and sexually active (had penetrative sex within the last 12 months).

### Sample size estimation

We selected condom use at last sex by HIV-positive AGYW as the indicator to guide our sample size calculation. Sample size estimation was undertaken with the intent of comparing differences in urban and rural populations. Data on condom use at last sex among sexually active HIV-positive AGYW in urban and rural settings were not readily available. However, the 2011 ZDHS [6] indicated that 17% of HIV-positive females aged 15–24 years who ever had sex reported condom use at last sexual intercourse in the past 12 months. These data were not disaggregated by residence (urban versus rural) and we estimated that 13% of HIV-positive AGYW in rural areas and 28% in urban areas would have used a condom at last sex. We also decided to recruit more participants in urban areas (ratio 2:1), since discussions with health care workers suggested that there were fewer HIV-positive AGYW in Mazowe compared to Harare. Using these parameters, we estimated a sample size of 360 (240 in urban areas; 120 in rural areas) based on a two-sided test, a significance level of 0.05, and 90% power to observe a difference of 15% in condom use between HIV-positive AGYW living in rural and urban areas.

### Data collection

Trained female interviewers collected the data using a structured questionnaire that was administered in Shona, the participants’ language. We interviewed HIV-positive AGYW to learn about their most recent male sexual partners’ sociodemographic characteristics and sexual behaviors. The survey instrument included questions adapted from questionnaires previously used in local and regional surveys. Questionnaire domains included sociodemographics and sexual behaviors of AGYW and their sex partners. Data were also collected to capture age at HIV status awareness, number of sexual partners in lifetime and in the past month preceding the survey, HIV status disclosure, reported mode of HIV infection, and adherence to treatment. To describe characteristics of participants’ most recent sex partners in the past 12 months preceding the survey, we collected sociodemographic characteristics of male sex partners, consensual or transactional sex, condom use, concurrent sexual relationships, identity of other sexual partners, and reported circumcision status. Interviews took place in a private room or space in all recruiting facilities.

### Data management and analysis

Completed questionnaires were checked for consistency and completeness on the day of the data collection, and errors were corrected in the field. To reduce entry errors and ensure a high level of data accuracy, we used a double-entry procedure to capture the data electronically. Questionnaires were entered into Microsoft Excel version 14.0, and data were exported to STATA SE version 13.0 (StataCorp, College Station, TX) for analysis. The characteristics of the study population were summarized using proportions for binary and categorical variables and means and medians (with 25^th^ and 75^th^ quantiles) for continuous variables. HIV-positive AGYWs’ and participants’ characteristics were compared by residence (rural vs. urban areas) using chi-square or Fisher exact tests for binary and categorical variables, and non-parametric Wilcoxon-Mann-Whitney test for continuous variables. Odds ratios were computed to measure association between an exposure and outcome of interest. Multivariate analysis was performed to identify predictors of ART adherence among HIV+ AGYW. Adherence was self-reported and defined as having never missed an ART dose. Participants were asked how often, on average, they miss taking their antiretroviral medications.

### Ethical considerations

Ethical approvals to conduct the study were obtained from the Medical Research Council of Zimbabwe (MRCZ/A/2076) and the John Snow, Inc. (JSI) Institutional Review Board for Protection of Research Subjects (IRB# 16–033). All participants provided written informed consent or assent (with parental consent).

## Results

### Sociodemographic characteristics of HIV-positive AGYW

Between September and November 2016, 360 HIV-positive AGYW took part in the study (Table 1). The median age of study participants was 22 years. Participants from rural areas were older than those recruited from urban areas (p < .05). Only 14% of HIV-positive AGYW did not complete primary school. Those from urban areas reported the highest level of schooling completed—specifically, from secondary school and beyond (74%, vs. 38% for rural participants). Very few participants (8%) were still attending school at the time of the survey; of these, the vast majority (86%) were from urban areas. The median age at first marriage (18 years) was similar for participants from both urban and rural areas. Thirty-eight percent of participants were currently married or living with their most recent male partner, but there were more married or cohabitating HIV-positive AGYW in rural (64%) than urban areas (25%) (p < .05). The vast majority of participants (75%) have lost at least one parent; more urban participants have lost both parents (45%) than those from rural areas (34%) (p < .05). In urban and rural areas, 50% (60/120) and 68% (13/19), respectively, of HIV-positive AGYW who reported being perinatally infected had lost both parents.

**Table 1:** Sociodemographic characteristics of HIV-positive adolescent girls and young women by location.

Variables	Overall (N = 360)	HIV+ AGYW (N = 240) Urban (Harare)	HIV+ AGYW (N = 120) Rural (Mazowe)	P value
**Age (years)**				< .01
Median (25^th^– 75^th^ percentile)	22 (19–23)	21 (19–23)	22 (20–24)	
**Highest level of schooling completed**				< .01
Did not complete primary school	47 (14%)	11 (5%)	36 (32%)	
Completed primary school	52 (16%)	23 (11%)	29 (26%)	
Some years of secondary school	86 (27%)	61 (29%)	25 (23%)	
Completed secondary school	126 (39%)	106 (50%)	20 (18%)	
University/Tertiary	12 (4%)	11 (5%)	1 (1%)	
**Currently attending school**				.02
Yes	29 (8%)	25 (11%)	4 (3%)	
No	323 (92%)	212 (89%)	111 (97%)	
**Ever been married**				< .01
Yes	226 (63%)	124 (52%)	102 (85%)	
No	134 (37%)	116 (48%)	18 (15%)	
**Age at first marriage (years)**				.4
Median (25^th^– 75^th^ percentile)	18 (16–19)	18 (16–19)	18 (17–19)	
**Current marital status**				< .01
Single	130 (36%)	113 (47%)	17 (14%)	
Married/living together	136 (38%)	59 (25%)	77 (64%)	
Divorced/separated/widowed	94 (26%)	68 (28%)	26 (22%)	
**Orphanhood**				.01
Both parents alive	90 (25%)	58 (24%)	32 (27%)	
Father deceased only	79 (22%)	42 (18%)	37 (31%)	
Mother deceased only	42 (12%)	32 (13%)	10 (8%)	
Both parents deceased	149 (41%)	108 (45%)	41 (34%)	

Not all totals add up because of missing data.

### HIV-related and sexual and reproductive characteristics of HIV positive AGYW

The median age at first sexual intercourse (17 years) was similar among both urban and rural participants (Table 2). The median number of male sexual partners HIV-positive AGYW had in their lifetime was 2 (IQR: 1–4). Participants in urban areas reported more partners both in their lifetime and in the last 12 months than their rural peers (p < .05). Forty-five percent (108/239) and 19% (46/239) of HIV-positive AGYW in urban areas had more than 2 partners in their lifetime and in the last 12 months, respectively. Thirty percent of participants (36/120) in rural areas had more than 2 partners in their lifetime. Only one rural participant reported more than 2 partners in the past 12 months.

**Table 2:** HIV-related and sexual and reproductive characteristics of HIV-positive adolescent girls and young women by location.

Variable	Overall (N = 360)	HIV+ AGYW (N = 240) Urban (Harare)	HIV+ AGYW (N = 120) Rural (Mazowe)	P value
**Age at first sexual intercourse (years)**				.2
Median (25^th^– 75^th^ percentile)	17 (15–19)	17 (15–19)	17 (15–18)	
**Number of sex partners in lifetime**				.01
Median (25^th^– 75^th^ percentile)	2 (1–4)	2 (1–4)	2 (1–3)	
**Number of sexual partners in the last 12 months**				< .01
Median (25^th^– 75^th^ percentile)	1 (1–1)	1 (1–2)	1 (1–1)	
**Age at first HIV status awareness (years)**				< .01
Median (25^th^– 75^th^ percentile)	17 (13.5–20)	16 (12–19)	19 (17–22)	
**Reported mode of HIV infection**				< .01
Perinatally	139 (39%)	120 (50%)	19 (16%)	
Sexually	193 (54%)	108 (45%)	85 (71%)	
Don’t know	28 (7%)	12 (5%)	16 (13%)	
**HIV status disclosure**				.03
Yes	335 (93%)	218 (91%)	117 (98%)	
No	25 (7%)	22 (9%)	3 (2%)	
**Awareness of participant’s HIV status by most recent sex partner**				< .01
Yes	238 (66%)	143 (60%)	95 (79%)	
No	122 (34%)	97 (40%)	25 (21%)	
**Mode of awareness of participant’s HIV status by most recent sex partner**	N = 238	N = 143	N = 95	.2
He had always known/someone told him	13 (6%)	11 (8%)	2 (2%)	
I told him	193 (81%)	114 (80%)	79 (83%)	
Others (CVCT/ member of ART support group)	32 (13%)	18 (12%)	14 (15%)	
**Antiretroviral treatment**				.2
Yes	339 (94%)	223(93%)	116 (97%)	
No	21 (6%)	17 (7%)	4 (3%)	
**Adherence to ART treatment**				< .01
Never missed a dose	259 (76%)	156 (70%)	103 (89%)	
Ever missed a dose	80 (24%)	67(30%)	13 (11%)	
**Ever been pregnant**				< .01
Yes	242 (67%)	137 (57%)	105 (88%)	
No	118 (33%)	103 (43%)	15 (12%)	
**Contraception use with most recent partner**				.3
Yes	267 (74%)	182 (76%)	85 (71%)	
No	93 (26%)	58 (24%)	35 (29%)	

Not all totals add up because of missing data.

The median age when participants learned for the first time about their HIV-positive status was 17 years (IQR: 13.5–20 years). HIV-positive AGYW in rural areas learned their HIV status much later, at a median age of 19 years (p < .05). The majority of participants were reportedly sexually infected (54%), with more rural participants heterosexually infected (71%) than those in urban areas (45%) (p < .05). Urban and rural perinatally infected participants knew about their HIV status at 13 and 15 years, respectively (p < .05). Among those heterosexually infected, urban participants learned about their HIV status earlier (at 19 years of age) than those in rural areas (20 years) (p < .05).

The majority of participants reported having disclosed their HIV status to someone; disclosure was more frequent among rural participants (98%) than their urban peers (91%) (p < .05). Rural participants were almost 4 times more likely to disclose their status to someone than their urban peers (OR = 3.9–95% CI: 1.1–20.9). Sixty-six percent of participants reported that their most recent male sexual partner knew about their HIV status, but only 54% (193/360) of all participants disclosed their status directly to their sexual partner. Male sexual partners of rural participants were more aware of their partner’s HIV status than those of urban participants (p < .05). Involuntary disclosure by a third person, couple voluntary counseling and testing, or attendance at an ART clinic or support group were other modalities by which male partners learned about participants’ HIV status.

Most participants were on ART (94%). A greater proportion of those in rural areas (89%) reported that they have never missed an ART dose as compared to their urban peers (70%) (p < .05). HIV-positive AGYW in rural areas were 2.6 times more likely to report never missing an ART dose as compared to their urban peers (adjusted OR = 2.59–95% CI: 1.16–5.58) after controlling for age, education level, reported mode of HIV infection and disclosure (Table 3). Adherence was also better among participants who reported to be heterosexually infected (adjusted OR = 1.99–95% CI: 1.03–3.84) as compared to those perinatally infected.

**Table 3:** Factors associated with reported ART adherence (never missing an ARV dose).

Variables	Crude OR	95% CI	Adjusted OR	95% CI
Current Age*	1.13	1.02–1.25	1.07	.94–1.22
Highest education level achieved				
Less than secondary level	-	-	-	-
Secondary level	.49	.25 –.95	.83	.38–1.82
University/Tertiary level	.33	.08–1.26	.63	.13–2.97
Reported Mode of HIV acquisition				
Perinatal transmission	-	-	-	-
Heterosexual transmission	2.72	1.62–4.54	1.99	1.03–3.84
Location				
Urban	-	-	-	-
Rural	3.40	1.78–6.47	2.59	1.16–5.80
Disclosure				
No	-	-	-	-
Yes	1.31	.49–3.52	0.92	.30–2.78
Current Marital status				
Single	-	-	-	-
Married	2.31	1.2–4.17	.85	.39–1.86
Other (widow/separated/divorced)	1.72	.90–3.28	.84	.39–1.82

*Current age as continuous variable.

Sixty-seven percent of participants have been pregnant at least once. More rural participants (88%) reported past pregnancy experience than their urban peers (OR = 5.2–95% CI: 2.8–9.5). Contraception (Long-acting reversible contraception, hormonal methods, barriers methods including male and female condoms, emergency contraception and sterilization) use with the most recent partner was frequent among participants (74%), and not significantly different in rural and urban participants.

### Most recent male sexual partners' characteristics and sexual activities as reported by HIV positive AGYW

Half of the participants reported that their most recent male sexual partner in the past 12 months was a current or former boyfriend (Table 4). Only urban participants reported having casual partners or male clients from sex work. The most recent male sexual partner in the past 12 months for rural participants was a current or former husband (68%), whereas for the majority of urban participants, he was a current or former boyfriend (59%).

**Table 4:** Sociodemographic and sexual behaviors of most recent male sexual partners of HIV-positive adolescent girls and young women by location.

Variable	Overall (N = 360)	HIV+ AGYW (N = 240) Urban (Harare)	HIV+ AGYW (N = 120) Rural (Mazowe)	P value
**Most recent sex partner in the past 12 months**				< .01
Current boyfriend	131 (36%)	104 (43%)	27 (23%)	
Current husband	129 (36%)	57 (24%)	72 (60%)	
Ex-boyfriend	49 (14%)	38 (16%)	11 (9%)	
Ex-husband	23 (6%)	13 (5%)	10 (8%)	
Male clients (sex work)/casual partners	28 (8%)	28 (12%)	0	
**Age of the most recent sex partner**				.4
Older	282 (78%)	183 (76%)	99 (82%)	
Younger	5 (2%)	4 (2%)	1 (1%)	
Same age	69 (19%)	49 (20%)	20 (17%)	
Don’t know	4 (1%)	4 (2%)	0	
**Age difference with the most recent sex partner**				.2
More than 10 years older	56 (20%)	30 (16%)	26 (26%)	
5 to 10 years older	114 (40%)	75 (41%)	39 (40%)	
Less than 5 years older	102 (36%)	71 (39%)	31 (31%)	
Don’t know	10 (4%)	7 (4%)	3 (3%)	
**Marital status of the most recent sex partner**				.01
Single	126 (35%)	108 (45%)	18 (15%)	
Married to/living together with me	140 (39%)	62 (26%)	78 (65%)	
Married to /living together with someone else	37 (10%)	26 (11%)	11 (9%)	
Widowed/separated/divorced	43 (12%)	32 (13%)	11 (9%)	
Don’t know	14 (4%)	12 (5%)	2 (2%)	
**Have children with the most recent partner**				.01
Yes	137 (49%)	66 (39%)	71 (65%)	
No	143 (51%)	104 (61%)	39 (35%)	
**Education level of most recent sex partner**				< .01
Did not complete primary school	8 (2%)	2 (1%)	6 (5%)	
Completed primary school	15 (4%)	5 (2%)	10 (8%)	
Some years of secondary school	39 (11%)	13 (5%)	26 (22%)	
Completed secondary school	180 (50%)	120 (50%)	60 (50%)	
University/Tertiary	69 (19%)	59 (25%)	10 (8%)	
Don’t know	49 (14%)	41 (17%)	8 (7%)	
**HIV status of the most recent sex partner**				< .01
Negative	79 (22%)	57 (24%)	22 (18%)	
Positive	125 (35%)	67 (28%)	58 (48%)	
Don’t know	156 (43%)	116 (48%)	40 (33%)	
**Place of first meeting with the most recent sex partner**				< .01
Nightlife/drinking venue	24 (7%)	23 (10%)	1 (1%)	
Public/commercial areas/bus station	223 (62%)	143 (60%)	80 (67%)	
Private events/places	51 (14%)	31 (13%)	20 (17%)	
Others (family/friend’s house/hidden site)	60 (17%)	42 (17%)	18 (15%)	
**Most recent sex partner’s concurrent relationship**				.7
I know he did not have sexual relations with other girls	68 (19%)	47 (20%)	21 (17%)	
I don’t believe he had sexual relationships with other girls	45 (12%)	26 (11%)	19 (16%)	
Yes, I know he had sexual relations with other girls	89 (25%)	59 (24%)	30 (25%)	
Yes, I believe he had sexual relations with other girls	64 (18%)	44 (18%)	20 (17%)	
Don’t know	94 (26%)	64 (27%)	30 (25%)	
**Identity of women in concurrent relationship with the most recent sex partner (N = 153)**	N = 153	N = 103	N = 50	.5
Other wives	29 (19%)	17 (17%)	12 (24%)	
Girlfriends	88 (57%)	61 (59%)	27 (54%)	
Sex workers	36 (24%)	25 (24%)	11 (22%)	
**Number of other partners the most recent partner had in the last 12 months**	N = 138	N = 89	N = 49	.2
Median (25^th^– 75^th^ percentile)	2 (1–3)	2 (1–3)	2 (1–3)	
**Condom use in the last 12 months with the most recent sex partner**				.01
Never	81 (23%)	60 (25%)	21 (17%)	
Sometimes	145 (40%)	84 (35%)	61 (51%)	
Always	134 (37%)	96 (40%)	38 (32%)	
**Condom use the last time you had sex with most recent sex partner**				.8
Yes	135 (38%)	89 (38%)	46 (39%)	
No	221 (62%)	148 (62%)	73 (61%)	
**Most recent sex partner’s alcohol use around time of sex**				.9
Yes	103 (29%)	69 (29%)	34 (28%)	
No	257 (71%)	171 (71%)	86 (72%)	
**Transactional sex in the past months with the most recent sex partner**				< .01
Yes	77 (21%)	61(25%)	16 (13%)	
No	283 (79%)	179 (75%)	104 (87%)	
**Circumcision status of the most recent sex partner**				< .01
Yes	92 (25%)	70 (29%)	22 (18%)	
No	219 (61%)	125 (52%)	94 (78%)	
Don’t Know	49 (14%)	45 (19%)	4 (4%)	

Not all totals add up because of missing data.

Male sexual partners were older than their female counterparts regardless of setting. Although not significant, there was a greater proportion of older partners in rural (82%) than urban areas (76%). The majority of male sexual partners (60%) were at least 5 years older than their female partners. A greater proportion of rural participants reported that their male sexual partner was more than 10 years older (26%, versus 16% among urban participants). Rural participants were 1.8 times more likely than their urban peers to report a partner more than 10 years older (OR = 1.8–95% CI = (1–3.2))

Most recent male sexual partners were married to or living with a participant (39%) or single (35%). The vast majority of rural participants were married to or living as married with their most recent male sexual partner (65%), whereas for urban participants, their most recent male sexual partner was single (45%). Ten percent of HIV-positive AGYW reported that they had a sexual relationship with married or cohabiting men.

Forty nine percent of study participants reported having a child with their most recent male sexual partner. Rural participants were almost three times more likely to report having a child with their most recent male sexual partner than their urban peers (OR = 2.8–95% CI: 1.7–4.7). Twenty-eight of 139 (20%) perinatally infected participants (24 urban and 4 rural) reported having a child with their most recent partner.

Only 2% percent of recent male sexual partners have not completed primary school. Most recent male sexual partners of urban participants (92%) were more educated (secondary school level and beyond) than those of their rural peers (65%) (p < .05).

The majority of participants (57%) knew about their most recent male sexual partner’s HIV status; but those in rural areas were more aware of their partner’s HIV status (66%) than their urban peers (52%). Among participants who had a child with their most recent male sex partner, 63% (86/137) did not know their own HIV status when they started having sex with that partner. Of those, 14% stated that their partner was HIV-negative, and 56% said that he was HIV-positive following a couple voluntary counseling and testing (CVCT) session. Most (55 out 86 (64%)) of these 86 positive AGYW were among rural participants; of these, 67% (37/55) knew about their partner’s status through CVCT. Among the 31 urban participants, 74% (n = 23) learned their partner’s HIV status through CVCT. Only 23% (81/360) of male partners disclosed their status, participants reported. There was no significant difference for disclosure by a male partner among rural (24/120) and urban participants (57/240). More HIV-positive AGYW in rural areas learned about their male partner’s HIV status (56/120) via CVCT than their urban peers (67/240) (OR = 2.2–95% CI = 1.4–3.5).

The majority of participants and their partners (62%) met for the first time in public or commercial locations like malls and bus stations. First meetings at drinking and or dancing venues were almost exclusively reported by urban participants (10%, or n = 23). Of these, 16 (70%) were engaged in sex work.

Forty-three percent of HIV-positive adolescent girls knew or believed that their most recent partner had sexual relationships with other girls. Their male partners were reportedly engaged in concurrent sexual relationships with other girlfriends (57%) and sex workers (24%). Nineteen percent of other concurrent sexual partners were other wives. Most recent male partners had a (median) number of 2 (IQR: 1–3) concurrent sexual partners. These findings were similar among sexual partners of both urban and rural HIV-positive AGYW. Thirty-nine percent (55/140) of participants who were married to or living with their most recent partners believed or knew that they had sexual relations with other girls (33/55 (60%)), other wives (6/55 (11%)), and sex workers (16/55 (29%)). Among HIV-positive AGYW who reported that their most recent sexual partners were single, 31% (39/126) believed or knew that they had other sexual partners, including girlfriends (37/39 or 95%) and sex workers (2/39 or 5%). There was no significant urban-rural disparity in terms of the identity of women in concurrent relationship with the most recent male sexual partner, although there were more polygamous male partners in rural than urban areas.

Consistent condom use in the last 12 months with male sexual partners was 37%. Urban participants reported more consistent condom use (40%) than their rural peers (32%). Condom use at last sex was also suboptimal (38%) but similar among sexual partners of both urban and rural participants. Condom use at last sex was significantly greater among single male partners (91/124 or 73%) than among male partners married or living with participants (78/138 or 57%) (OR = 2.1–95% CI: 1.2–3.5). It was not significantly greater than among male partners living or married to someone else (22/37 or 59%).

Alcohol consumption around the time of sexual intercourse was frequent (29%) but did not significantly differ among partners of both urban and rural participants. It was more prevalent among male clients (61% or 17/28) of HIV-positive AGYW who were involved in sex work than among current/ex-boyfriend (24% or 44/180) and current/ex-husband (28% or 44/180) of HIV-positive AGYW.

Twenty-one percent of participants reported transactional sex in the past 12 months with their most recent sexual partner. More urban participants were engaged in transactional sex than their rural peers. They were twice as likely to get involved in transactional sex in the last 12 months preceding the survey than their rural peers (OR = 2.2–95% CI: 1.2–4). Single (30/130 or 23%) and previously married women (widowed, divorced, and separated) (39/94 or 41%) were more involved in transactional sex than those who were married (8/136 or 6%). None of the participants with university- or tertiary-level education reported being engaged in transactional sex with their most recent partner.

**Medical circumcision status of the sex partner.** Only 25% of participants reported that their most recent male sexual partner was circumcised. However, circumcision was significantly more prevalent among sexual partners of urban participants (29%) than among those of their rural peers (18%). If we considered only participants who knew the circumcision status of their male partners, male partners of urban participants were twice more likely to be circumcised than those of their rural peers (OR = 2.3–95% CI: 1.3–4.1).

## Discussion

Understanding the sociodemographic characteristics of HIV-positive AGYW, their male sex partners, and their sexual behaviors could support the development and implementation of more relevant prevention interventions among both people living with HIV and their partners. Clearer understanding also could identify risk factors that might explain the high risk of HIV infection among AGYW in Zimbabwe.

In our cross-sectional study, HIV-positive AGYW from rural areas were older than their urban peers, although we targeted those between 15 and 24 years old in both settings. This finding could be explained by the low participation of 15–19-year-old participants, especially in rural areas, who did not want to disclose to their parents that they were sexually active by participating in the study. There was a huge disparity in education among our study participants. HIV-positive AGYW in rural areas were less likely to be in school at the time of the survey and to have completed a higher level of education than those in urban areas. These findings were similar to those from the last ZDHS and the comparative analysis of AGYW in three settings in Kenya and South Africa [2, 10].

Age at first marriage was similar for urban and rural participants, although in Zimbabwe, AGYW in rural areas tend to get married earlier (2 years earlier) than their urban peers [2]. More rural participants were married as compared to their urban peers. Similar disparity in marital status among urban and rural AGYW have been reported in Zimbabwe and in many sub-Saharan African countries [2, 10, 38, 39].

Our study population was greatly affected by orphanhood, which might be partially explained by the death of at least one HIV-positive parent (87%) among those who reported being perinatally infected. According to the latest ZDHS, among children aged 10–14 years, those with at least one deceased parent were more likely than children with both parents living to have HIV [2]. This association could partially explain the urban-rural disparity when comparing participants who lost both parents.

The median age at first sexual intercourse was similar among urban and rural participants, and lower (by 1 year) than the median age at first marriage, revealing the occurrence of premarital sex in both groups. Early sexual debut in AGYW is common, not only in Zimbabwe but also in other sub-Saharan African countries [2, 29, 40, 41]. Overall, HIV-positive AGYW in our study reported a small number of male sexual partners over their lifetime; participants in urban areas reported having a greater number of male partners in their lifetime and in the last 12 months. Similar disparities were reported by the 2015 ZDHS, which showed that 2.2% and 0.8% of 15–24-year-old women in urban and rural areas had more than 2 partners in the past 12 months, respectively. It is worth mentioning that the number of partners reported by participants could be affected by underreporting of multiple or concurrent partnerships [42–44].

HIV-positive AGYW recruited from urban clinics were aware of their HIV status earlier than those from rural clinics. This observation could be explained by the greater number of perinatally infected AGYW recruited in urban clinics, who might learn their HIV status much earlier than heterosexually infected adolescents in rural areas.

More than a half of HIV positive AGYW reported being heterosexually infected which reflects the nature of the HIV epidemic in Zimbabwe [45, 46]. The greater number of perinatally infected participants in urban areas could be explained by our recruitment strategy and also a reluctance for participation by younger adolescents in rural areas.

The vast majority of participants have disclosed their HIV status to someone, but slightly more than half have directly shared their HIV status with their most recent sex partner. The greater rate of HIV status disclosure among rural participants could be explained by their greater likelihood to be tested during pregnancy and to be encouraged to disclose their status to their partner. The overall low prevalence of disclosure to sexual partners in Zimbabwe could be explained by fear of physical abuse, divorce, stigma, discrimination, and rejection among HIV-positive AGYW [47–49]. In a study exploring the sexual behavior of adolescents living with HIV in Zimbabwe, only 24% of those who were sexually active had disclosed their serostatus to their partners before sexual intercourse [50]. Reluctance to disclose HIV status among adolescents living with HIV is not unique to Zimbabwe and has been reported in many others countries in sub- Saharan Africa [51–54].

Almost all participants were on antiretroviral treatment, and most reported never having missed a dose. Adherence was significantly greater among rural than urban participants. This could be explained by increased partner support for ART after HIV status disclosure or CVCT, which has been identified as a correlate of ART adherence [31, 51, 55, 56]. Perinatally infected AGYW were more likely to report having missed ART dose during their treatment than those who reportedly were sexually infected. Adherence among perinatally infected adolescent is more challenging as they have to face very early on a lifelong treatment with its associated side effects in addition to other structural factors [57]. Our result also could be explained by the fact that adherence is often shared between caregivers and young adolescents and has been described as suboptimal when the caregiver is not the biological parent [58]. In our study, we have more participants who lost both parents in urban than rural areas. It is also worth mentioning that we have not collected data related to other factors that have been associated with adherence like stigma, distance from health facilities, stock out of medications and health care workers’ attitude towards clients.

As expected more participants in rural areas reported being pregnant at least once in their lifetime. This disparity is similar to findings reported among AGYW in Zimbabwe where 27% of rural teenagers have begun childbearing as compared to 10% of urban teenagers [2]. There was no disparity for contraceptive use but a little more than a quarter of study participants did not use any contraception measure with their most recent partner which could be explained by a desire for childbearing [59]. Close to 50% of study participants had a child with their most recent partner.

For HIV-positive AGYW recruited in urban health facilities, the most recent male sexual partner in the 12 months preceding our survey was most often a current or former boyfriend. Among those recruited in rural facilities, the most recent partner in the last 12 months was a current or former husband. The spousal partner was also reported as the main sexual male partner type by AGYW in rural Kenya, but not in rural South Africa. This disparity may be explained by the different sociocultural context of marriage in both countries [10].

Only a small number of HIV-positive AGYW, mainly from urban areas, were involved in sex work and they were either divorced or single. Young HIV-positive sex workers are at substantial risk of disease progression if they do not engage in HIV prevention and treatment, which could contribute to the worsening of the local HIV epidemic [60–62].

Many HIV-positive AGYW in Zimbabwe are engaged in age-disparate relationships. They have sexual relationships with much older men, a sexual mixing pattern that has been reported frequently in Zimbabwe and in Africa in general [3, 10, 63, 64]. Relationships with male partners more than 10 years older was more prevalent among rural than urban participants. Similar findings were reported in a comparative study assessing the characteristics of male partners of AGYW in three settings of the Determined, Resilient, Empowered, AIDS-free, Mentored and Safe (DREAMS) Project [10].

Most recent male partners of HIV-positive AGYW in rural areas were married to or living with them as married. In urban areas, the most recent male partner was single. This situation mirrors the marriage pattern in the general population in Zimbabwe, where urban and or educated women marry later than rural women [2].

Most of the male partners of HIV-positive AGYW have achieved a certain level of education. The gap in educational attainment, specifically at secondary level and beyond, between participants’ male partners from urban and rural areas reflects the gap in educational attainment in the general population in Zimbabwe between rural and urban populations [2].

Slightly more than half of participants knew their male partner’s HIV status. Very few male partners disclosed their status directly to their female partners; CVCT played an important role for HIV status disclosure among partners, and occurred more frequently among rural than urban study participants. CVCT during pregnancy for PMTCT could be a likely explanation of this disparity, since there were more married participants and pregnancies recorded among rural than urban AGYW. Male partners’ reluctance to disclose status or limited self-disclosure has been explained by the fear of rejection, stigma, public exposure, fatalism, and secrecy regarding sexual matters [48, 51, 53, 65].

Public and commercial venues like malls, shopping centers, and bus and taxi stations were important meeting places for sexual partners. Drinking and dancing venues were mainly patronized by partners of urban participants who were involved in sex work. Meeting places could represent good venues to display or disseminate HIV prevention messages and available intervention services [3, 66–68].

Both HIV-positive AGYW and their most recent partners were involved in concurrent sexual partnerships with married or single partners, regardless of study area. Concurrent sexual partnership did not significantly differ among urban and rural participants. Concurrency has been recognized in Zimbabwe as an important factor fueling or sustaining the HIV epidemic [8, 9, 63, 69, 70]. Concurrent sexual relationship can put both AGYW and married couples at increased risk of contracting or transmitting HIV. In his study on concurrent sexual partnerships among Zimbabweans, Mugweni et al. identified four possible types of concurrent sexual relationships: sex worker, casual partner, regular girlfriend, or informal polygyny [8]. One out of 10 study participants acknowledged having sexual relationship with a married or cohabiting male partner. Forty-six percent of participants who reported having relationships with married or cohabiting partners were engaged in sex work.

Consistent condom use was sub-optimal among HIV-positive AGYW and their most recent male partners. In-depth interviews with some of our participants revealed that low condom use was due to their lack of confidence about suggesting condom use to their partners, their low HIV risk perception due to poor comprehensive HIV knowledge, and intimate partner violence [30]. Low condom use among married couples and HIV-positive women has been reported in previous studies in Zimbabwe [71–73]. The lack of significant urban-rural disparity on condom use was not expected, and we think that it might be due in part to social desirability bias.

More urban participants and their most recent male sexual partners were engaged in transactional sex. Numerous factors could explain this finding, including poverty, need to access material goods and money, or gift exchange in romantic relationships [74–76].

Alcohol consumption has often been associated with intimate partner violence and HIV infection [77–80]. In our study, we did not find a significant difference in alcohol consumption around the time of sexual intercourse among male partners of urban and rural participants, but it was more frequent among male clients of HIV-positive AGYW who were involved in commercial sex, which suggests the need for prevention interventions at drinking venues or meeting places [3, 66, 67].

Male circumcision is an effective strategy that is currently being scaled up in Zimbabwe for reducing HIV infection among men. However, we found a low circumcision rate among the most recent partners of HIV-positive AGYW in our study, and an urban-rural disparity similar to that found in the 2015 DHS findings [2]. Circumcision prevalence was higher among partners of urban than rural participants. Male circumcision in the general population is still suboptimal, with 18% and 12% of urban and rural men aged 15–49 years reporting having been circumcised [2], respectively. Low uptake of male circumcision in Zimbabwe has been linked to several factors, including fear of pain, fear of poor wound healing, knowledge gaps regarding male medical circumcision, and cultural beliefs and practices [81, 82].

Our study has some limitations. We had difficulty recruiting 15–19-year-old HIV-positive AGYW in rural areas. Reportedly, some mothers in rural areas did not want their daughters to participate because they had not yet disclosed their status to their daughters. Also, some AGYW under the age of 18 years did not want their parents to know that they were engaging in sexual activity.

We also did not include male partners in our study as participants. We felt that AGYW are better placed to talk about their sexual behaviors and their male partners. In addition, trying to find male partners of these HIV positive AGYW posed the risk of unintentional HIV status disclosure. However, studies have reported social desirability bias in sexual behavior reporting among both men and women [83, 84]. Men tend to report more sexual and casual partnerships than women. In Clark’s study in Kisumu Kenya [83], men and women tended to agree about whether men had other non-marital partners but disagreed about women’s non-marital partners.

We did not have enough cases of perinatally infected AGYW in rural areas to stratify our analysis by age group, areas, or mode of infection (sexual vs. maternal transmission). The same limitation applies to analysis of lifetime sexual partners. Only a limited number of HIV-positive AGYW reported more than one partner, so some of our results should be interpreted with caution.

All the data we collected were self-reported; thus we cannot exclude the possibility of desirability and recall bias. Several studies have shown that women tend to underreport early sexual activity and the number of their sexual partners [83, 85]. We were unable to use a computer-assisted survey instrument, which has been shown to increase reporting of sensitive behaviors [44]. Nevertheless, our study results were similar to findings from other studies in Zimbabwe that investigated risky sexual behaviors among AGYW. Our results might not be generalizable to other sub-Saharan African settings, but they indicate a need to evaluate and/or strengthen existing prevention programs for AGYW living with HIV.

## Conclusions

Our study showed that most recent male sexual partners of HIV-positive AGYW recruited in Harare were current or former boyfriends, older, single, with at least a high-school level of education, and less likely to disclose their HIV status to partners. They were more likely to engage in concurrent relationship with sex workers, be circumcised, use condoms more consistently in the last 12 months preceding the survey, drink alcohol around the time of sexual intercourse, especially with female sex workers, and to engage in transactional sex. Most recent male sexual partners of HIV-positive AGYW recruited in rural clinics in Mazowe district were a current or former husband, likely to be more than 10 years older than their female partners and to have a child with them. There were less educated than partners of urban HIV positive AGYW, and more likely to engage in CVCT, be polygamous, use condoms inconsistently in the last 12 months preceding the survey, and less likely to be circumcised.

In Zimbabwe, prevention programs should target all male sexual partners of HIV-positive AGYW; but in urban areas, interventions should focus on partners of HIV positive AGYW who have had more than one sexual partner in their lifetime or have engaged in sex work. HIV education and information activities on HIV testing, as well as prevention interventions, should be constantly promoted at meeting places [67]. In rural areas, HIV prevention activities promoting couple or index case testing should be strengthened to reach the sexual partners of AGYW, particularly those who are HIV-positive [86]. Adherence to ART should be reinforced at every interaction between service providers and HIV-positive AGYW. Consistent condom use, and sexually transmitted infection screening should also be recommended, both to HIV-positive AGYW facing challenges with ART adherence and their male sexual partners [72]

Structural interventions to keep AGYW in school (e.g., school fee programs), income-generating activities, and interventions to address challenges associated with harmful gender norms, orphanhood, and early marriage should be part of a comprehensive prevention package for all AGYW, regardless of their HIV status.

Our study findings showed important urban-rural disparities among HIV-positive AGYW and their most recent male sexual partners. These findings will likely furnish an important complement to several other studies looking at characterizing male partners of AGYW in SSA in general, and Zimbabwe in particular.

## Supporting information

S1 File (XLSX)

S1 Questionnaire (DOC)
