# Supplementary material for: Urban-rural disparity in sociodemographic characteristics and sexual behaviors of HIV-positive adolescent girls and young women and their perspectives on their male sexual partners: A cross-sectional study in Zimbabwe
Source: PLoS One. 2020 Apr 23;15(4):e0230823. doi: 10.1371/journal.pone.0230823 (PMC7179911; doi:10.1371/journal.pone.0230823)
Supplement: S1 Questionnaire — (DOC) [file pone.0230823.s002.doc]

**Adolescent Girls and Young Women Study Questionnaire**

| **Geographic and Site Information** | |
| --- | --- |
|  |  |
| COUNTRY _____________________­______________________ |_____|_____|  pROVINCE/REGION NAME ________________________ |_____|_____|  DISTRICT NAME ___________________________________ |_____|_____|  FACILITY NAME ___________________________________ |_____|_____|  Facility/PARTICIPANT CODE _____________________ | Type of area:  URBAN 1  PERI-URBAN 2  RURAL 3 |
| INTERVIEWER NAME: _____________________________________  FIELD SUPERVISOR NAME:________________________________  DATE OF INTERVIEW:________________________________  TIME INTERVIEW STARTED: ____________________  TIME INTERVIEW FINISHED: _____________________ | |

**Note: Please, ensure that this section is properly filled in before starting the survey.**

| ***I will start by asking some questions about you.*** | | | | |
| --- | --- | --- | --- | --- |
| **NO.** | **QUESTIONS AND FILTERS** | | **RESPONSES CODES/SKIPS** | |
|  | How old were you at your last birthday? *(If unsure, please give your best guess).* | Years old: | |  |
|  | Have you ever attended school?  *ONE RESPONSE ONLY* | No................................................................................1  Yes...............................................................................2 | | N🡪F06 |
|  | Are you currently attending school?  *ONE RESPONSE ONLY* | No................................................................................1  Yes...............................................................................2 | | Y🡪 F05 |
|  | What is the highest level of schooling you have completed?  *ONE RESPONSE ONLY* | Less than primary........................................................1  Completed Primary......................................................2  Some Secondary (F1 – F3)..........................................3  Completed Secondary (F4/O-Level) …………………..4  Completed A-Level…….…………………………………5  Tertiary ………………..……………….………...............6 | | 🡪F06 |
|  | What is your current level of school?  *ONE RESPONSE ONLY* | Less than primary........................................................1  Completed Primary......................................................2  Some Secondary (F1 – F3)..........................................3  Completed Secondary (F4/O-Level) …………………..4  Completed A-Level…….…………………………………5  Tertiary ………………..……………….………...............6 | |  |
|  | What is your religion?  *ONE RESPONSE ONLY* | Catholic........................................................................1  Anglican.......................................................................2  Lutheran.......................................................................3  Methodist.....................................................................4  Baptist..........................................................................5  Presbyterian.................................................................6  Apostolic (Marange or Masowe)..................................7  Pentecostal..................................................................8  Jehovah’s Witness………………………………………9  Muslim..........................................................................10  African traditional religion.............................................11  Other (Specify)______________________________12 | |  |

| F07 | What is your ethnic group?  *ONE RESPONSE ONLY* | Shona……………………………………………………..1  Ndebele…………………………………………………..2  Other………………………………………………………3  (Specify) ___________________________________ |  |
| --- | --- | --- | --- |
| F08 | Have you ever been married or ever lived with a male as if married?  *ONE RESPONSE ONLY*  *NOTE:* For the purposes of this survey, *“*Married” refers to a relationship in which there has been payment of lobola and/or the woman was wedded through either traditional or religious ceremony. | No................................................................................1  Yes...............................................................................2 | N🡪F10 |
| F09 | How old were you when you first got married or lived with someone as if married? *(If unsure, please give your best guess).* | | Years old: | | --- | |  |
| F10 | What is your current marital status?  *ONE RESPONSE ONLY* | Married (i.e. lobola paid/wedded) ……………………..1  Living together but not married (i.e. lobola not  paid/not wedded).........................................................2  Widowed ……………………………………………........3  Divorced …………………………………………............4  Separated…………………………………………...........5  Never married...............................................................6 |  |
| F11 | Where do you **mostly** get your money?  *ONE RESPONSE ONLY* | Formal job (f/t salaried)..................................................1  Informal job (self-employed)..........................................2  Other paid job (part-time casual)…................................3  Selling/hawking..............................................................4  Farming/gardening………………………….....................5  Panning (gold/diamond) ................................................6  Parent/caregiver.............................................................7  Husband.........................................................................8  Boyfriend/romantic partner ………………......................9  Older man who gave me something in exchange  for sex………………………………………………………10  Male clients (sex work) ……………………………………11  Other………………………………………………………..12    (Specify)____________________________________ |  |

| F12 | Who **usually** decides how the money you earn/receive will be used?  *ONE RESPONSE ONLY* | I decide…………………………………………………..1  My husband decides………………………………......2  My boyfriend/romantic partner decides………………3  My parent/caregiver decides....……..…………….......4  Someone else decides………………………………… 5  (Specify)___________________________________  I decide jointly with someone………………………….. 6  (Specify)___________________________________  Don’t know.................................................................. 98 |  |
| --- | --- | --- | --- |
| F13 | Altogether, how many days, weeks or months were you away from home in the **LAST 12 months**?  *ONE RESPONSE ONLY* | Days______................................................................1  Weeks______..............................................................2  Months______.............................................................3 |  |
| F14 | Are your parents living?  *ONE RESPONSE ONLY* | Both living....................................................................1  Father deceased..........................................................2  Mother deceased.........................................................3  Both deceased.............................................................4  Don’t know.................................................................. 98 |  |
| F15 | Who do you currently live with?  ***DON'T PROMPT, CHECK ALL THAT APPLY*** | No one (I live alone).....................................................1  Mother…......................................................................2  Step-Mother………………………………………………3  Father……………………………………………………..4  Step-Father……………………………………………….5  Elder brother/sister.......................................................6  Younger brother/sister…………………………………..7  Caregiver/relative.........................................................8  Husband.......................................................................9  Boyfriend/romantic partner.....................………………10  My Child/Children……..…………………………………11  Neighbor.....................................................................12  Friend/colleague..........................................................13  Other…………………………………………………….. 14  (Specify)___________________________________ |  |

| F16 | Who is the head of your household?  *ONE RESPONSE ONLY* | I am the head of household.........................................1  Someone else……………………………………………. 2  (Specify)____________________________________ | | |  |
| --- | --- | --- | --- | --- | --- |
| ***Now, we would like to ask you questions about HIV testing and treatment. Some questions may make you feel uncomfortable. Please remember that you do not need to answer any question that makes you feel uncomfortable. Anything you tell me will be confidential, and I am not going to judge you for any responses you provide*** | | | | | |
| F17 | How old were you when you were first told you have HIV? *(If unsure, please give your best guess).* | Age: | | |  |
| F18 | In your view, how did you get HIV?  ***DO NOT PROMPT***  ***ONE RESPONSE ONLY*** | From my mother..............................................................1  From a sexual act with someone I knew - We agreed  to have sex ………….......................................................2  From a sexual act with someone I knew – We did not  agree to have sex; used physical force or coercion….…3  From a stranger/rapist.....................................................4  From a blood transfusion.................................................5  From a contaminated sharp object...................................6  Other (Specify)______________________________ 7  Don’t know.......................................................................98 | | | If not a sexual act  🡪F20 |
| F19 | Who was the person who gave you HIV from a sexual act?  *ONE RESPONSE ONLY* | Husband………………………………………………….1  Boyfriend/Romantic partner…………………………….2  Older man who gave me something in exchange  for sex ……………………………………………….……3  Male client (sex work)…………………………………… 4  Relative……………………………………………………5  Stranger……………………………………………………6  Other (specify) …_____________________________7 | | |  |
| F20 | Where were you tested and told you have HIV?  *ONE RESPONSE ONLY* | New Start center (HCT)...............................................1  Mobile testing...............................................................2  Home testing................................................................3  Treatment center.........................................................4  ANC clinic....................................................................5  Other health facility......................................................6  Other (Specify)______________________________7  Don’t know.................................................................98 | | |  |
|  |  |  | | |  |
| F21 | Have you ever told anyone the results of your HIV test(s)?  *ONE RESPONSE ONLY* | No (please provide.......................................................1 reasons____________________________________  Yes.............................................................................2 | | | N🡪F23 |
| F22 | I am going to read out a list of people you could have told. Please tell me if you have told them about your HIV status..   1. Father 2. Mother 3. Brother(s) 4. Sister(s) 5. Other family members/relatives 6. Caregiver 7. Husband 8. Boyfriend/romantic partner 9. Older man who gave me something in exchange for sex 10. Male client (sex work) 11. A friend who is not someone   attending my support group   1. A health worker 2. A pastor or priest 3. My employer 4. Support group leader 5. Other (Specify) __________............... | |  |  |  |  | | --- | --- | --- | --- | |  | N | Y | N/A | | A. Father | 1 | 2 | 100 | | B. Mother | 1 | 2 | 100 | | C. Brother(s) | 1 | 2 | 100 | | D. Sister(s) | 1 | 2 | 100 | | E. Other family members/relatives | 1 | 2 | 100 | | F. Other Caregiver | 1 | 2 | 100 | | G. Husband | 1 | 2 | 100 | | H. Boyfriend/romantic partner | 1 | 2 | 100 | | I. Older man who gave me something in exchange for sex | 1 | 2 | 100 | | J. Male client (sex work) | 1 | 2 | 100 | | K. A friend who is not someone attending my support group | 1 | 2 | 100 | | L. A health worker | 1 | 2 | 100 | | M. A priest or pastor | 1 | 2 | 100 | | N. Your employer | 1 | 2 | 100 | | O. Support group leader | 1 | 2 | 100 | | P. Other (Specify) __________.......... | 1 | 2 | 100 | | | |  |
| F23 | Are you currently taking ARVs? *ARVs are tablets that reduce the amount of HIV in your body.*  *ONE RESPONSE ONLY* | No..............................................................................1  Yes.............................................................................2  Don’t know.................................................................98 | | | N🡪F25 |
| F24 | On average, how often do you miss taking your ARVs?  *ONE RESPONSE ONLY* | Never……………………………………………………..1  Once a day.................................................................2  Twice a week............................................................3  Once a week.............................................................4  Once every two weeks..............................................5  Once a month............................................................6  Other……………………………………………………..7    (Specify) __________________________________ | | |  |
| F25 | Apart from medical care, which other services do you receive because of your HIV status?  ***READ THE LIST ALOUD, CHECK ALL THAT APPLY*** | None….................................................................................1  Support from Child Adherence Treatment Supporter (CAT).2  Support from community health worker................................3  Support group services.........................................................4  Home based care..................................................................5  Financial assistance..............................................................6  Food assistance....................................................................7  Nutrition counseling...............................................................8  Other………………………………………………………..........9  (Specify) ___________________________________ | | |  |
| F26 | Have you ever had any of the following symptoms on your private parts: vaginal discharge with an unusual smell, pain when urinating, sore on genitals, warts on genitals, itching, burning or swelling in the genital area?  *ONE RESPONSE ONLY* | No................................................................................1  Yes...............................................................................2. | | |  |
| F27 | Have you ever been pregnant?  *ONE RESPONSE ONLY* | No................................................................................1  Yes...............................................................................2 | | | N🡪F29 |
| F28 | How many live births have you ever had? | Number | | |  |
| F29 | Have you ever tried any of the following (substances)?  ***READ THE LIST ALOUD, CHECK ALL THAT APPLY***     |  |  | Never tried | Tried once or twice | Use some-  Times | Use regularly/  Often (many times) | | --- | --- | --- | --- | --- | --- | | a | Mbanje (marijuana) | 1 | 2 | 3 | 4 | | b | Glue | 1 | 2 | 3 | 4 | | c | Beer | 1 | 2 | 3 | 4 | | d | Wine (not at church) | 1 | 2 | 3 | 4 | | e | Alcohol (other than beer or wine) | 1 | 2 | 3 | 4 | | | | | |
| ***The next questions are about sexual activity. These questions may make you feel uncomfortable. We are asking about these things so that we will be able to fully understand how we should develop HIV prevention programs among adolescent girls and young women. We would like very much to have you help us by telling us about yourself as much as you can. Remember that your name will never be linked to the information that you give us, and we will not judge you because of any answers you provide. By sex we mean vaginal or anal.*** | | | | | |
| Now I’d like to ask you about the first time you had sex. | | | | | |
| F30 | How old were you when you had sex for the very **first time?** *(If unsure, please give your best guess).*  *When thinking about the “first time”, please consider any sexual experience (intercourse) including whether you agreed, or you were pressured, or you were physically forced/raped, with anyone you knew or did not know before.* | | | Years old: | | --- |   Don’t know................................................................ | |  |
| F31 | The **FIRST TIME** you had sex, was it because you had agreed?  *ONE RESPONSE ONLY* | | Yes, we both agreed to have sex…………….….1  No, I hadn’t agreed; I was pressured/coerced….2  No, I was physically forced/raped………………. 3 | |  |
| F32 | Who was the person you had sex with the very **first time**?  *ONE RESPONSE ONLY* | | Current boyfriend/Romantic partner........……....1  Current husband…………………………………..2  Ex-boyfriend/romantic partner……….................3  Ex-husband………………………………………..4  Older man who gave me something in  exchange for sex …………………………………5  Male Client (sex work)..………………………….6  Father…………………........................................7  Step father……………………….........................8  Brother…………………………………………......9  Uncle…………………………………………….....10  Male friend/schoolmate…………........................11  Male teacher.......................................................12  Male police/Security person................................13  Male employer....................................................14  Male neighbor………………...............................15  Male community/religious leader..........…….......16  Male stranger………………………………..........17  Other male (Specify) ___________________ 18  Don’t know.........................................................98 | |  |
| F33 | Was this person older than you, younger than you, or about the same age?  *ONE RESPONSE ONLY* | | | Older..................................................................1  Younger............................................................2  About same age...............................................3  Don’t know........................................................98 | If younger or about the same age  🡪F35 |
| F34 | Was this person more than 10 years older than you, 5-10 years older or less than 5 years older?  *ONE RESPONSE ONLY* | | | More than 10 years older…..............................1  5-10 years older……………………….…...........2  Less than 5 years older….................................3  Don’t know........................................................98 |  |
| F35 | What was this person’s marital status when you had sex for the very **FIRST TIME**? | | | Married to me (i.e. lobola paid/wedded) …………1  Living together with me but not married (i.e. lobola not paid/not wedded)............................................2  Married to someone else………………………......3  Living together with someone else…………….....4  Widowed ………………………………………........5  Divorced ………………………………………….....6  Separated……………………………………….......7  Never married.......................................................8  Don’t know..........................................................98 |  |
| F36 | What was his educational level when you had sex for the very **first time?**  *ONE RESPONSE ONLY* | | | Less than primary.................................................1  Completed Primary...............................................2  Some Secondary (F1 – F3)..................................3  Completed Secondary (F4/O-Level) …………...4  Completed A-Level…….………………………….5  Tertiary …………………..…….………................6  Don’t know.........................................................98 |  |
| F37 | Where did he **mostly** get his money?  *ONE RESPONSE ONLY* | | | Formal job (f/t salaried).....................................1  Informal job (self-employed) .............................2  Other paid job (part-time casual)……………….3  Selling/hawking.................................................4  Farming/gardening…………………………........5  Panning (gold/diamonds)..................................6  Driving truck/bus/commuter omnibus...............7  Parent/caregiver…………………………………8  Other…………………………………………….. 9  (Specify)____________________________  Don’t know.......................................................98 |  |
| F38 | Was the person you had sex with the very **FIRST TIME** from the same community where you were living? | | No…………………………………………………1  Yes………………………………………………..2  Don’t know......................................................98 | |  |
| F39 | Where did you meet for the **first** time?  That is to say – where were you introduced, or got to know him for the first time?  *ONE RESPONSE ONLY* | | | Nightlife/drinking venue: eating, drinking,  dancing and sleeping sites...............................1  Open/transport-related: transport, public, commercial areas..............................................2  Events/private: (example: concert, sports  event, party, wedding).......................................3  Hidden sites (example: abandoned yard,  field, bush)………………………………………...4  His house…………….…………………………....5  His friend’s house………………………………...6  Other…………………………………………….....7  (Specify)____________________________  Don’t know.........................................................98 |  |
| F40 | Where did you have sex for the **FIRST** time? | | | Nightlife/drinking venue: eating, drinking,  dancing and sleeping sites...............................1  Open/transport-related: transport, public, commercial areas..............................................2  Events/private: (example: concert, sports  event, party, wedding).......................................3  Hidden sites (example: abandoned yard,  field, bush)………………………………………...4  His house…………….…………………………....5  His friend’s house………………………………...6  Other…………………………………………….....7  (Specify)____________________________  Don’t know.........................................................98 |  |
| F41 | When did you **first** meet him - how long ago (from today)?  *ONE RESPONSE ONLY* | | | Days______........................................................1  Weeks_____........................................................2  Months _____.......................................................3  Years______........................................................4 |  |
| F42 | How long had you known him before you had sex?  *ONE RESPONSE ONLY* | | | Hours______........................................................0  Days______..........................................................1  Weeks______.......................................................2  Months ______....................................................3  Years______........................................................4 |  |
| F43 | Do you know or believe that he had sexual relations with other girls or women at this time?  *ONE RESPONSE ONLY* | | | No, I know he did not….......................................1  No, I don’t believe/think he did……………….......2  Yes, I know he did................................................3  Yes, I believe/think he did....................................4  Don’t know..........................................................98 | N🡪F46 |
| F44 | Who are these other women?  *CHECK ALL THAT APPLY* | | | Other wives..............................………………..1  Girlfriends/'small houses'.................................2  Sex workers/bar girls...............………………..3  Other……………………………………………..4    (Specify) ___________________ |  |
| F45 | If you took a guess, how many other partners do you think he had the **FIRST TIME** you had sex (apart from you)? | | | Number of other partners: |  |
| F46 | The **first time** you had sex, did you or the man use a condom from start to finish?  *ONE RESPONSE ONLY*  *Note: “you” refers to the respondent using a female condom; “the man” refers to the man using a male condom.* | | | No…................................................................1  Yes...................................................................2  Don’t know.......................................................98 | N🡪F48 |
| F47 | Where did you or this person obtain the condoms?  *ONE RESPONSE ONLY* | | | Shop/bar............................................................1  Health facility......................................................2  Community health worker...................................3  Peer educator..................................................4  Other...............................................................5  (Specify) _____________________________  Don’t know.......................................................98 |  |
| F48 | The **FIRST TIME** you had sex, did the man drink any alcohol beforehand or between rounds of sex?  *ONE RESPONSE ONLY* | | | No....................................................................1  Yes..................................................................2  Don’t know......................................................98 |  |
| F49 | The **FIRST TIME** you had sex, did you drink any alcohol beforehand or between rounds of sex?  *ONE RESPONSE ONLY* | | | No....................................................................1  Yes..................................................................2  Don’t know.......................................................98 |  |
| F50 | Was this person circumcised?  *ONE RESPONSE ONLY* | | | No....................................................................1  Yes..................................................................2  Don’t know......................................................98 |  |
| F51 | Were you already HIV positive when you had sex the very **first time**?  *ONE RESPONSE ONLY* | | | No....................................................................1  Yes..................................................................2  Don’t know......................................................98 |  |
| F52 | Did the person you had sex with the very **first time** know your HIV status?  *ONE RESPONSE ONLY* | | | No....................................................................1  Yes..................................................................2  Don’t know......................................................98 | N🡪F54 |
| F53 | How did he get to know your HIV status?  *ONE RESPONSE ONLY* | | | Had always known/someone else told him........1  I told him............................................................2  Other………………………………………………. 3  (Specify) ______________________________  Don’t know.........................................................98 |  |
| F54 | What was the HIV status of this person?  *ONE RESPONSE ONLY*  *Probe as needed to clarify whether the respondent “knows” about the partner’s HIV status, or “thinks” she knows the partner’s HIV status (i.e. he didn’t tell her, but she thinks she has an idea about the partner’s HIV status).* | | | **NEGATIVE**  I think this person was HIV-negative………….1  This person told me he was HIV-negative........2  He was HIV-negative; we tested together……3  **POSITIVE**  I think this person was HIV-positive……………4  He told me he was HIV-positive.......................5  He was HIV-positive; we tested together……..6  Don’t know......................................................98 |  |
| F55 | Did this person give you anything for having sex with him the very **FIRST TIME**?  *ONE RESPONSE ONLY* | | | No....................................................................1  Yes...................................................................2 | N🡪F57 |
| F56 | What did he give you?  *ONE RESPONSE ONLY* | | | Cash……………………………………………....1  In-Kind..............................................................2  Cash and in-kind………………………………....3  IF IN-KIND, PLEASE SPECIFY:  ________________________________________________________________________________________________________________________ |  |

| **Thank you for telling me about this first time you had sex. Now, I would like to ask you some questions about other people with whom you have had sex, if there have been any others.** | | | | |
| --- | --- | --- | --- | --- |
| F57 | How many people have you had sex with in your **lifetime?** *(If unsure, please give your best guess).*  *Must be completed, given eligibility criteria* | Number of Persons: | | |
| F58 | How many people have you had sex within the **past 12 months**?  *Must be completed, given eligibility criteria* | Number of Persons: | | |
| **IF THE RESPONDENT HAS HAD MORE THAN ONE LIFETIME SEXUAL PARTNER, USE THE TABLE BELOW TO CONTINUE COLLECTING INFORMATON ABOUT PARTNERS AFTER THE FIRST PARTNER, AND UNTIL THE MOST RECENT SEXUAL PARTNER IN THE PAST 12 MONTHS (ALSO TO BE COMPLETED IN THE QUESTIONNAIRE).**  **USE THE TABLE BELOW FOR ALL PARTNERS OTHER THAN FIRST SEXUAL PARTNER, AND MOST RECENT SEXUAL PARTNER IN PAST 12 MONTHS.** | | | | |
|  |  | 2^ND^ partner | 3^rd^ partner | 4^th^ partner |
| M01 | Who was the second/third/fourth person you had sex with?  Current boyfriend/Romantic partner...... 1  Current husband.……….…………… 2    Ex-boyfriend/romantic partner….……... 3  Ex-husband...……………….…………… 4  Older man who gave me something in  exchange for sex…………………………….5  Male Client (sex work)……………..………..6  Father….….…………............................. 7  Step father…………………….…........... 8  Brother………………………………….. 9    Uncle…………………….……….. 10  Male friend/schoolmate…………….…....... 11  Male teacher............................................ 12  Male police/Security person.................... 13  Male employer........................................ 14  Male neighbor………………................... 15  Male community/religious leader............ 16  Male stranger………..…………….......... 17  Other male…………....…………………… 18  (Specify)______________________________    Don’t know…………………………………… 98  *ONE RESPONSE ONLY* |  |  |  |
| M02 | Was this person older than you, younger than you, or about the same age?  Older................................................................... 1  Younger.............................................................. 2  About same age................................................... 3  Don’t know........................................................ 98  *ONE RESPONSE ONLY*  *IF YOUNGER OR ABOUT THE SAME AGE* 🡪M04 |  |  |  |
| M03 | Was this person more than 10 years older than you, 5-10 years older or less than 5 years older?  More than 10 years older…................................. 1  5-10 years older….……………….….................... 2  Less than 5 years older...….................................. 3  Don’t know...........................................................98  *ONE RESPONSE ONLY* |  |  |  |
| M04 | What was this person’s marital status?  Married to me……………………………………...............1  Living together with me but not married..........................2  Married to someone else…………………………….........3  Living together with someone else………………….........4  Widowed ……………………………………………............5  Divorced …………………………………………................6  Separated…………………………………………..............7  Single (never married).....................................................8  Don’t know …………………………………………….......98  *ONE RESPONSE ONLY* |  |  |  |
| M05 | What was his education level?  Less than primary............................................................1  Completed Primary........................................................2  Some Secondary (F1 – F3)...........................................3  Completed Secondary (F4/O-Level) …………………...4  Completed A-Level…….…………………………………5  Tertiary ………………..……………….………...............6  Don’t know ……………………………………………....98 |  |  |  |
| M06 | Where did he **mostly** get his money?  Formal job (f/t salaried)................................................1  Informal job (self-employed) ........................................2  Other paid job (part-time casual)………………............3  Selling/hawking............................................................4  Farming/gardening…………………………...................5  Panning (gold/diamonds).............................................6  Driving truck/bus/commuter omnibus..........................7  Parent/caregiver…………………………………...........8  Other……………………………………………..............9  (Specify)____________________________  Don’t know..................................................................98  *ONE RESPONSE ONLY* |  |  |  |
| M07 | Was this person from the same community where you were living?  No…………………………………………………...........1  Yes…………………………………………………..........2  Don’t know…………………………………………........98 |  |  |  |
| M08 | Where did you meet this person for the **first** time?  *That is to say – where were you introduced, or got to know him for the first time*?  Nightlife/drinking venue: eating, drinking,  dancing and sleeping sites.............................................1  Open/transport-related: transport, public,  commercial areas............................................................2  Events/private: (example: concert, sports event, party, wedding).........................................................................3  Hidden sites (example: abandoned yard, field, bush).....4  His house…………….………………………….................5  His friend’s house…………………………………….........6  Other (Specify)____________________________........7  *ONE RESPONSE ONLY* |  |  |  |
| M09 | How long had you known him before you first had sex? *(unprompted)*  *ONE RESPONSE ONLY*  Hours______..................................................................1  Days______....................................................................2  Weeks______.................................................................3  Months _____..................................................................4  Years_____.....................................................................5 | Hours______......1  Days______........2  Weeks______.....3  Months _____.....4  Years_____........5 | Hours______......1  Days______........2  Weeks______.....3  Months _____.....4  Years_____........5 | Hours______......1  Days______........2  Weeks______.....3  Months _____.....4  Years_____........5 |
| M10 | Do you know or believe that he had sexual relations with other girls or women at the same time that he was having sex with you?  No, I know he did not……………………….....................1  No, I don’t believe/think he did…………….....................2  Yes, I know he did………………………………...............3  Yes, I believe/think he did……………………..................4  Don’t know………………………………………...............98 | 1🡪M13 | 1🡪M13 | 1🡪M13 |
| M11 | Who were these other women?  Other wives..............................………………...............1  Girlfriends/'small houses'..............................................2  Sex workers/bar girls...............………………...............3  Other……………………………………………...............4    (Specify) _____________________________  *CHECK ALL THAT APPLY* |  |  |  |
| M12 | If you took a guess, how many other partners do you think he had (apart from you)? | Number of other partners: | Number of other partners: | Number of other partners: |
| M13 | If you had sex with this person only once, did you or he use a condom from start to finish?  No.............................................................................1  Yes...........................................................................2  Don’t know...............................................................98  *ONE RESPONSE ONLY*  *SKIP TO M18 IF THE RESPONDENT HAD SEX WITH THIS PARTNER MORE THAN ONCE.*  *Note: “you” refers to the respondent using a female condom; “he” refers to the man using a male condom.* |  |  |  |

| M14 | If you had sex with this person more than once, how often did you or he use a condom from start to finish?  Never………………………………………………......1  Rarely……………………………………………….....2  Some of the time …………………………….............3  Most of the time………………………………............4  Always ……………………………………………........5  *ONE RESPONSE ONLY*  *Note: “you” refers to the respondent using a female condom; “he” refers to the man using a male condom.* |  |  |  |
| --- | --- | --- | --- | --- |

| M15 | Before or between rounds of sex did the person you had sex with ever drink any alcohol?  No…………………………………………………….1  Yes…………………………………………………..2  Don’t know…………………………………………98  *ONE RESPONSE ONLY* |  |  |  |
| --- | --- | --- | --- | --- |
| M16 | Before or between rounds of sex with this person, did you ever drink any alcohol?  No…………………………………………………….1  Yes…………………………………………………..2  Don’t know…………………………………………98  *ONE RESPONSE ONLY* |  |  |  |
| M17 | Was this person circumcised?  No…………………………………………………….1  Yes…………………………………………………..2  Don’t know…………………………………………98  *ONE RESPONSE ONLY* |  |  |  |

| M18 | Did this person know your HIV status?  No…………………………………………………….1  Yes…………………………………………………..2  Don’t know…………………………………………98  *ONE RESPONSE ONLY* |  |  |  |
| --- | --- | --- | --- | --- |
| M19 | What was the HIV status of this person?  *ONE RESPONSE ONLY*  *Probe as needed to clarify whether the respondent “knows” about the partner’s HIV status, or “thinks” she knows the partner’s HIV status (i.e. he didn’t tell her, but she thinks she has an idea about the partner’s HIV status).*  **NEGATIVE**  I think this person was HIV-negative………….1  This person told me he was HIV-negative........2  He was HIV-negative; we tested together……3  **POSITIVE**  I think this person was HIV-positive……………4  He told me he was HIV-positive.......................5  He was HIV-positive; we tested together……..6  Don’t know......................................................98 |  |  |  |
| M20 | The **FIRST TIME** you had sex with this person, was it because you had agreed?  Yes, we both agreed to have sex…………….….1  No, I hadn’t agreed; I was pressured/coerced….2  No, I was physically forced/raped………………. 3 |  |  |  |

| M21 | Did this ever person give you anything for having sex with him?  No....................................................................1  Yes..................................................................2  *ONE RESPONSE ONLY* |  |  |  |
| --- | --- | --- | --- | --- |
| M22 | What did he give you?  *ONE RESPONSE ONLY*  Cash……………………………………………....1  In-Kind..............................................................2  Cash and in-kind………………………………....3 |  |  |  |
|  | IF IN-KIND, PLEASE SPECIFY:  PARTNER 2: ______________________________________________________________________________________  PARTNER 3: ______________________________________________________________________________________  PARTNER 4: ______________________________________________________________________________________ | | | |

| Thank you for sharing with me this information about your previous sex partners. You told me earlier that you have had XX partnerS in the **PAST 12 MONTHS**. I would like to ask some questions about **the most recent sex partner in the past 12 months** (this could be your current partner). | | | | | |
| --- | --- | --- | --- | --- | --- |
| ***Think about the most recent male you had sex with in the past 12 months.*** | | | | | |
| F59 | Who is the most recent male you had sex with **IN THE PAST 12 MONTHS**?  *ONE RESPONSE ONLY* | | Current boyfriend/Romantic partner........……....1  Current husband…………………………………..2  Ex-boyfriend/romantic partner……….................3  Ex-husband………………………………………..4  Older man who gave me something in  exchange for sex …………………………………5  Male Client (sex work)..………………………….6  Father………………….......................................7  Step father……………………….........................8  Brother…………………………………………......9  Uncle……………………………………………....10  Male friend/schoolmate………………..…….......11  Male teacher......................................................12  Male police/Security person...............................13  Male employer....................................................14  Male neighbor………………...............................15  Male community/religious leader..........…….......16  Male stranger………………………………..........17  Other male (Specify) ___________________ 18  Don’t know.........................................................98 |  | |
| F60 | Is this person older than you, younger than you, or about the same age?  *ONE RESPONSE ONLY* | | Older..................................................................1  Younger.............................................................2  About same age................................................3  Don’t know........................................................98 | If younger or about the same age  🡪F62 | |
| F61 | Is this person more than 10 years older than you, 5-10 years older or less than 5 years older?  *ONE RESPONSE ONLY* | | More than 10 years older….............................1  5-10 years older……………………….…..........2  Less than 5 years older…................................3  Don’t know.......................................................98 |  | |
| F62 | What is this person’s marital status? | | Married to me (i.e. lobola paid/wedded) ……...1  Living together with me but not married (i.e. lobola not paid/not wedded)........................................2  Married to someone else………………………..3  Living together with someone else…………….4  Widowed ………………………………………...5  Divorced …………………………………………6  Separated………………………………………..7  Never married..................................................8 |  | |
| F63 | **Not applicable (if respondent has never**  **been pregnant or given birth)**  Do you have any children with this person?  *ONE RESPONSE ONLY* | | No....................................................................1  Yes..................................................................2 |  | |
| F64 | What is his educational level?  *ONE RESPONSE ONLY* | | Less than primary..............................................1  Completed Primary............................................2  Some Secondary (F1 – F3)...............................3  Completed Secondary (F4/O-Level) …………...4  Completed A-Level…….…………………………5  Tertiary …………………..…….………...............6  Don’t know........................................................98 |  | |
| F65 | Where does he **mostly** get his money?  *ONE RESPONSE ONLY* | | Formal job (f/t salaried).....................................1  Informal job (self-employed) .............................2  Other paid job (part-time casual)……………….3  Selling/hawking.................................................4  Farming/gardening…………………………........5  Panning (gold/diamonds)..................................6  Driving truck/bus/commuter omnibus................7  Parent/caregiver…………………………………..8  Other…………………………………………….. ..9  (Specify)____________________________  Don’t know........................................................98 |  | |
| F66 | Is this person from the same community where you live? | | No…………………………………………………….1  Yes……………………………………………………2 |  | |
| F67 | Where did you meet for the **first** time?  *ONE RESPONSE ONLY* | | Nightlife/drinking venue: eating, drinking,  dancing and sleeping sites...................................1  Open/transport-related: transport, public, commercial areas.................................................2  Events/private: (example: concert, sports  event, party, wedding)..........................................3  Hidden sites (example: abandoned yard,  field, bush)………………………………………......4  His house…………….………………………….......5  His friend’s house………………………………......6  Other…………………………………………….......7  (Specify)____________________________ |  | |
| F68 | Where did you have sex for the **FIRST** time? | | Nightlife/drinking venue: eating, drinking,  dancing and sleeping sites..................................1  Open/transport-related: transport, public, commercial areas................................................2  Events/private: (example: concert, sports  event, party, wedding).........................................3  Hidden sites (example: abandoned yard,  field, bush)………………………………………....4  His house…………….…………………………....5  His friend’s house………………………………....6  Other……………………………………………......7  (Specify)____________________________  Don’t know.........................................................98 |  | |
| F69 | When did you **first** meet him - how long ago (from today)?  *ONE RESPONSE ONLY* | | Days______........................................................1  Weeks_____........................................................2  Months _____.......................................................3  Years______........................................................4 |  | |
| F70 | How long had you known him before you first had sex? *(unprompted)*  *ONE RESPONSE ONLY* | | Hours______........................................................0  Days______..........................................................1  Weeks______.......................................................2  Months ______....................................................3  Years______........................................................4 |  | |
| Thank you for the information you’ve provided so far. Now I’m going to ask you some questions about how frequently you’ve had sex with this person. | | | | | |
| F71 | When was the last time you had sex with him?  *ONE RESPONSE ONLY* | | Hours______........................................................0  Days______..........................................................1  Weeks______.......................................................2  Months ______....................................................3 |  | |
| F72 | ***(Interviewer: For next three questions, probe for number of rounds***  How many rounds of sex have you had with this person in the **past week**?  *(If unsure, please give your best guess).* | | Number of rounds: |  | |
| F73 | How many rounds of sex have you had with this person in the **past month?**  *(If unsure, please give your best guess).* | | Number of rounds: |  | |
| F74 | How many rounds of sex have you had with this person in the **past 12 months**?  *(If unsure, please give your best guess).*  *Must be completed, given eligibility criteria* | | Number of rounds: |  | |
| F75 | Do you know or believe that he currently has sexual relations with other girls or women?  *ONE RESPONSE ONLY* | | No, I know he did not…...................................1  No, I don’t believe/think he did……………..…2  Yes, I know he did...........................................3  Yes, I believe/think he did................................4  Don’t know......................................................98 | N🡪F78 | |
| F76 | Who are these other women?  *ONE RESPONSE ONLY* | | Other wives..............................………………..1  Girlfriends/'small houses'..................................2  Sex workers/bar girls...............………………...3  Other (Specify) ___________________..........4 |  | |
| F77 | If you took a guess, how many other partners do you think he has had in the last 12 months (apart from you)? | | Number of other partners: |  | |
| F78 | In the **LAST 12 months** when you had sex with this person, how often did you use a condom from start to finish?  *ONE RESPONSE ONLY* | | Never……………………………………………...1  Rarely................................................................2  Some of the time...............................................3  Most of the time.................................................4  Always...............................................................5 |  | |
| F79 | The last time you had sex with this person, did you or he use a condom from start to finish?  *ONE RESPONSE ONLY*  *Note: “you” refers to the respondent using a female condom; “he” refers to the man using a male condom.* | | No....................................................................1  Yes..................................................................2  Don’t know......................................................98 |  | |
| F80 | Between the two of you, who **usually** brings the condoms?  *ONE RESPONSE ONLY* | | Myself...............................................................1  My partner........................................................2  Both of us.........................................................3 |  | |
| F81 | Where do you or this person **usually** obtain the condoms?  *ONE RESPONSE ONLY* | | Shop/bar...........................................................1  Health facility.....................................................2  Community health worker..................................3  Peer educator....................................................4  Other.................................................................5  (Specify) _____________________________  Don’t know.......................................................98 |  | |
| F82 | Before or between rounds of **last sex** did the person you had sex with drink any alcohol?  *ONE RESPONSE ONLY* | | No.....................................................................1  Yes....................................................................2  Don’t know.......................................................98 |  | |
| F83 | Before or between rounds of **last sex** did you drink any alcohol?  *ONE RESPONSE ONLY* | | No.....................................................................1  Yes....................................................................2  Don’t know.......................................................98 |  | |
| F84 | Is this person circumcised?  *ONE RESPONSE ONLY* | | No.....................................................................1  Yes....................................................................2  Don’t know.......................................................98 |  | |
| F85 | Did you already know your HIV status when you started to have sex with this partner  *ONE RESPONSE ONLY* | | No.....................................................................1  Yes....................................................................2  Don’t know.......................................................98 |  | |
| F86 | Does this person know your HIV status?  *ONE RESPONSE ONLY* | | No.....................................................................1  Yes....................................................................2  Don’t know.......................................................98 | N🡪F88 | |
| F87 | How did he get to know?  *ONE RESPONSE ONLY* | | Had always known/someone else told him........1  I told him............................................................2  Other……………………………………………….3  (Specify) ______________________________  Don’t know.........................................................98 |  | |
| F88 | What is the HIV status of this person?  *ONE RESPONSE ONLY*  *Probe as needed to clarify whether the respondent “knows” about the partner’s HIV status, or “thinks” she knows the partner’s HIV status (i.e. he didn’t tell her, but she thinks she has an idea about the partner’s HIV status).* | | **NEGATIVE**  I think this person was HIV-negative………….1  This person told me he was HIV-negative........2  He was HIV-negative; we tested together……3  **POSITIVE**  I think this person was HIV-positive……………4  He told me he was HIV-positive.......................5  He was HIV-positive; we tested together……..6  Don’t know......................................................98 |  | |
| F89 | In the **past 12 months**, did you ever have sex with this person because he would give you something for having sex with him?  *ONE RESPONSE ONLY* | | No.....................................................................1  Yes....................................................................2 | N🡪F91 | |
| F90 | What did he give you?  *ONE RESPONSE ONLY* | | Cash……………………………………………....1  In-Kind...............................................................2  Cash and in-kind………………………………....3  IF IN-KIND, PLEASE SPECIFY:  ________________________________________________________________________________________________________________________ |  | |
| F91 | In the **past 12 months**, did you ever have sex with this person because he physically forced you/raped you?  *ONE RESPONSE ONLY* | | No.....................................................................1  Yes....................................................................2 |  | |
| F92 | In the **past 12 months**, did you ever have sex with this person because he pressured you into having sex through sex harassment, threats or tricks?  *ONE RESPONSE ONLY* | | No.....................................................................1  Yes....................................................................2 |  | |
| F93 | Are you and/or this person currently doing something or using any method to delay or avoid you getting pregnant?  *ONE RESPONSE ONLY* | | No.....................................................................1  Yes....................................................................2  Don’t know........................................................98 | N🡪F95 | |
| F94 | Which method are you and/or this person using?  *DON'T PROMPT, CHECK ALL THAT APPLY* | | Female sterilization…...………………................1  Male sterilization……….………………...............2  IUD...........…………………………………….......3  Injectables (e.g. Depo Provera)………………...4  Implants (e.g Jadelle, Norplant) …………….....5  Pill……………………………………………........6  Male condom……………………………….........7  Female condom…………………………….........8  Diaphragm…………………………………..........9  Foam/jelly…………………………………..........10  LAM…………………………………………….....11  Rhythm method…………………………............12  Withdrawal……………………………….............13  Other method………………………………….....14  (Specify) ____________________________  Don’t know.........................................................98 |  | |
| ***We would like to ask you some questions about using condoms. We realize that some people may be comfortable using condoms with certain types of partners, but not with others.*** | | | | | |
| F95 | Do you feel comfortable using condom **if suggested** by each of the following? | |  |  |  | | | --- | --- | --- | --- | |  | N | Y | N/A | | A. Husband/partner | 1 | 2 | 100 | | B. Fiancée | 1 | 2 | 100 | | C. Boyfriend | 1 | 2 | 100 | | D. Male friend | 1 | 2 | 100 | | E. Men who pay for sex | 1 | 2 | 100 | | F. Stranger | 1 | 2 | 100 | | G. Other (Specify) __________ | 1 | 2 | 100 | | | |  |
| F96 | Do you feel comfortable **suggesting** condom use to each of the following? | |  |  |  | | | --- | --- | --- | --- | |  | N | Y | N/A | | A. Husband/partner | 1 | 2 | 100 | | B. Fiancée | 1 | 2 | 100 | | C. Boyfriend | 1 | 2 | 100 | | D. Male friend | 1 | 2 | 100 | | E. Men who pay for sex | 1 | 2 | 100 | | F. Stranger | 1 | 2 | 100 | | G. Other (Specify) __________ | 1 | 2 | 100 | | | |  |

| ***I’m going to read some phrases about how confident you are in suggesting condom use with a sex partner. For each statement, please tell me if you strongly disagree, disagree, agree, or strongly agree.*** | | | |
| --- | --- | --- | --- |
| F97 | I feel confident in my ability to suggest using condoms with a new partner.  *ONE RESPONSE ONLY* | Strongly disagree........................................................1  Disagree.....................................................................2  Agree ..........................................................................3  Strongly agree............................................................4 |  |
| F98 | I feel confident I could suggest using a condom without making my partner feel like I think he might have a disease.  *ONE RESPONSE ONLY* | Strongly disagree........................................................1  Disagree.....................................................................2  Agree ..........................................................................3  Strongly agree............................................................4 |  |
| F99 | I feel confident in my ability to persuade any partner to accept using a condom when we have sex.  *ONE RESPONSE ONLY* | Strongly disagree........................................................1  Disagree.....................................................................2  Agree ..........................................................................3  Strongly agree............................................................4 |  |
| F100 | I wouldn’t feel confident suggesting using condoms with a new partner because I would be afraid he would think I’ve had several sexual partners.  *ONE RESPONSE ONLY* | Strongly disagree........................................................1  Disagree.....................................................................2  Agree ..........................................................................3  Strongly agree............................................................4 |  |
| F101 | I wouldn’t feel confident suggesting using condoms with a new partner because I would be afraid he would think I have an STI.  *ONE RESPONSE ONLY* | Strongly disagree........................................................1  Disagree.....................................................................2  Agree ..........................................................................3  Strongly agree............................................................4 |  |
| F102 | I wouldn’t feel confident suggesting using condoms with a new partner because I would be afraid he would think I thought they had an STI.  *ONE RESPONSE ONLY* | Strongly disagree........................................................1  Disagree.....................................................................2  Agree ..........................................................................3  Strongly agree............................................................4 |  |
| F103 | I feel confident that I would remember to use a condom even after I have been drinking/smoking mbange.  *ONE RESPONSE ONLY* | Strongly disagree........................................................1  Disagree.....................................................................2  Agree ..........................................................................3  Strongly agree............................................................4 |  |
| F104 | I feel confident that I would be able to buy or get condoms myself if I needed to use them.  *ONE RESPONSE ONLY* | Strongly disagree........................................................1  Disagree.....................................................................2  Agree ..........................................................................3  Strongly agree............................................................4 |  |
| ***Relationship communication***  ***Please think about the most recent sex partner you’ve had in the past 12 months, and tell me if you strongly disagree, disagree, agree, or strongly agree with the following statements.*** | | | |
| F105 | He becomes jealous when I wear things that make me look too beautiful.  *ONE RESPONSE ONLY* | Strongly disagree........................................................1  Disagree.....................................................................2  Agree ..........................................................................3  Strongly agree............................................................4 |  |
| F106 | He does whatever he wants, even if I don’t want him to do it.  *ONE RESPONSE ONLY* | Strongly disagree........................................................1  Disagree.....................................................................2  Agree ..........................................................................3  Strongly agree............................................................4 |  |
| F107 | I can talk with him about anything, including sex, without fearing he will get angry or annoyed.  *ONE RESPONSE ONLY* | Strongly disagree........................................................1  Disagree.....................................................................2  Agree ..........................................................................3  Strongly agree............................................................4 |  |
| F108 | When he and I disagree, we end up doing what he wants most of the time.  *ONE RESPONSE ONLY* | Strongly disagree........................................................1  Disagree.....................................................................2  Agree ..........................................................................3  Strongly agree............................................................4 |  |
| F109 | He has more control than me over whether or not we have sex..  *ONE RESPONSE ONLY* | Strongly disagree........................................................1  Disagree.....................................................................2  Agree ..........................................................................3  Strongly agree............................................................4 |  |
| F110 | He has more control than me over whether or not we use condoms.  *ONE RESPONSE ONLY* | Strongly disagree........................................................1  Disagree.....................................................................2  Agree ..........................................................................3  Strongly agree............................................................4 |  |
| F111 | He lets me know that I am not his only wife/girlfriend.  *ONE RESPONSE ONLY* | Strongly disagree........................................................1  Disagree.....................................................................2  Agree ..........................................................................3  Strongly agree............................................................4 |  |
| F112 | If we have problems in our relationship with each other, we usually find a way to discuss them and find solutions together.  *ONE RESPONSE ONLY* | Strongly disagree........................................................1  Disagree.....................................................................2  Agree ..........................................................................3  Strongly agree............................................................4 |  |
| ***Communication around HIV & HIV prevention services***  ***Please think about the most recent sex partner you’ve had in the past 12 months when answering the following questions.*** | | | |
| F113 | In the **past 12 months**, which of the following have you been able to, or would you have been able to do so if and when you wanted: | |  | N | Y | | --- | --- | --- | | A. Talk openly with him about sex? | 1 | 2 | | B. Disclose your HIV status to him? | 1 | 2 | | C. Advise him to take an HIV test? | 1 | 2 | | D. Advise him to undergo medical male circumcision for HIV prevention? | 1 | 2 | | E. Advise him to take medication that minimizes chances of contracting HIV? | 1 | 2 | |  |

**Thank you very much for sharing this information with me during the interview. Can I answer any questions you might have for me?**
